# A Multi-Institutional Study on the Efficacy and Safety of Wearing a Custom-Made Compression Elastic Garment for 6 Months for Klippel–Trenaunay Syndrome with Venous Malformation

**DOI:** 10.3390/jcm14134808

**Published:** 2025-07-07

**Authors:** Miho Noguchi, Sadanori Akita, Fumio Nagai, Tadashi Nomura, Tsuyoshi Morishita, Shunsuke Yuzuriha

**Affiliations:** 1 Department of Plastic and Reconstructive Surgery, Shinshu University School of Medicine, Matsumoto 390-8621, Japan; yuzuriha@shinshu-u.ac.jp; 2 Department of Bioregulation and Pharmacological Medicine, Fukushima Medical University, Fukushima 960-1247, Japan; 3 Department of Plastic and Reconstructive Surgery, Tamaki Aozora Hospital, Tokushima 779-3125, Japan; 4 Department of Plastic and Reconstructive Surgery, Toyama University Hospital, Toyama 930-0152, Japan; 5 Department of Plastic Surgery, Kobe University Graduate School of Medicine, Kobe 650-0017, Japan; 6 Department of Plastic and Reconstructive Surgery, Aichi Children’s Health and Medical Center, Obu 474-8710, Japan

**Keywords:** Klippel–Trenaunay syndrome, venous malformation, compression therapy, custom-made elastic garment, venous stasis

## Abstract

**Background:** Klippel–Trenaunay syndrome (KTS) is a congenital vascular malformation syndrome characterized by low-flow vascular anomalies, including venous malformation (VM) and lymphatic involvement. These anomalies may lead to limb asymmetry due to soft tissue and/or bone overgrowth. Compression therapy using elastic garments is considered a conservative and minimally invasive first-line treatment option for KTS. However, the benefits of compression therapy for low-flow vascular malformations, particularly limb VMs, have not been sufficiently evaluated. This prospective, multi-center study assessed the efficacy and safety of compression therapy for KTS with VM. **Methods:** After measuring the affected limb, a custom-made elastic garment providing 30 mmHg of compression was manufactured (THUASNE, France). A total of 20 patients (7 male, 13 female; mean age: 10.9 years) underwent compression therapy for 26 weeks at four nationwide institutions in Japan. The primary outcome was the change in lower limb circumference. Secondary outcomes included pain, modified Rankin Scale (mRS) score, body water content, vital signs, changes in garment elasticity, and adverse events. **Results:** All 20 patients completed the study. At the study endpoint, the circumference ratio of the affected to unaffected limbs was significantly reduced at the superior end of the tibial tuberosity (*p* = 0.02) and the thinnest part of the ankle (*p* < 0.001). The elastic force of the garment declined by approximately 50% over 26 weeks. No serious adverse events related to the intervention were reported. **Conclusions:** Compression therapy using a custom-made elastic garment appears to be a safe and effective approach for managing limb overgrowth in patients with KTS and VM. To maintain the therapeutic effect, garment replacement is recommended at least every six months.

## 1. Introduction

Klippel–Trenaunay syndrome (KTS) is a congenital vascular malformation syndrome involving low-flow vascular anomalies such as capillary malformations, venous malformations (VMs), and lymphatic malformations. These abnormalities can result in limb asymmetry and disfigurement due to the overgrowth of soft tissue and/or bone [1]. KTS arises from vascular developmental defects during fetal growth and is classified within the PIK3CA-related overgrowth spectrum [2]. Limb overgrowth is typically observed at birth or during infancy and progressively worsens with age. Associated complications include pain, skin ulceration, infection, and lymphorrhea [3]. Currently, no definitive treatment exists for KTS, and patients require lifelong symptomatic management involving sclerotherapy, embolization, excision, compression therapy, and pharmacotherapy [1]. For controlling limb overgrowth, the available options include lower extremity orthoses, corrective orthopedic surgery [4], and pharmacological agents such as mTOR inhibitors [2].

Compression therapy has been reported to alleviate symptoms of low-flow vascular malformations in the extremities, including pain, swelling, ulceration, and coagulopathy [5]. As a conservative and minimally invasive treatment, it is considered an appropriate first-line option for KTS. Similarly to VMs, lower extremity varicosities can cause venous stasis, and compression therapy is effective in relieving associated symptoms such as pain and edema [6]. Despite its clinical use, the quantitative and qualitative benefits of compression therapy for limb vascular malformations in KTS remain poorly characterized. Additionally, standard ready-made elastic garments often do not fit well in patients with KTS due to limb overgrowth and anatomical deformities.

This study aimed to objectively evaluate the efficacy and safety of compression therapy using custom-made elastic garments in patients with KTS and VM. Since KTS is a syndrome of combined vascular malformation, its clinical character is mixed and patients can show various clinical features depending on which low-flow vascular malformation mainly develops. Our prior pilot study suggested that compression therapy seemed to be more effective in patients with KTS showing the apparent clinical character of VM than other KTS patients, and we emphasized this by adding the term VM in this report. The primary outcome was the change in lower limb circumference. Secondary outcomes included pain, modified Rankin Scale (mRS) score for disability, body water content, vital signs, and adverse events. Changes in garment elasticity were also assessed to evaluate material durability.

## 2. Materials and Methods

### 2.1. Study Design and Participants

This prospective, single-arm, multi-center interventional study was conducted between August 2021 and January 2022 at four institutions in Japan: Shinshu University School of Medicine, Fukuoka Children’s Hospital, Kobe University School of Medicine, and Aichi Children’s Health and Medical Center. The study was registered in the Japan Registry of Clinical Trials (jRCT1030210271), with recruitment commencing on 24 August 2021.

Eligible participants were diagnosed with KTS involving unilateral venous malformation (VM) of a lower limb according to diagnostic criteria from the Japan Intractable Diseases Information Center (Table 1). All diagnoses were confirmed by magnetic resonance imaging prior to enrollment. The inclusion criteria were as follows: age ≥ 2 years, modified Rankin Scale (mRS) grade 1–4, and the ability to walk independently or with assistance. The exclusion criteria included bilateral KTS, recent treatment within 12 weeks (e.g., sclerotherapy or surgery), planned interventions during the study period, known allergy to garment materials, regular use of diuretics, recent heart failure within the past year, or any condition incompatible with study participation.

From August 2021 to January 2022, we enrolled participants for this multi-center, single-arm, prospective interventional study at four centers across Japan: Shinshu University School of Medicine, Fukuoka Children’s Hospital, Kobe University School of Medicine, and Aichi Children’s Health and Medical Center. This investigation was registered in the Japan Registry of Clinical Trials listed the International Clinical Trials Registry Platform (JRPN-jRCT1030210271), with recruitment starting on the same day (24th August 2021). The patient population was defined as those with KTS with the VM of one lower leg meeting the diagnostic criteria of the Japan Intractable Diseases Information Center (Table 1). All VM cases were evaluated by magnetic resonance imaging at diagnosis prior to the study. The inclusion criteria were age ≥ 2 years, mRS scale grade 1–4, and being able to walk even with an assistance device. The exclusion criteria included having a KTS lesion in both lower limbs; being <12 weeks after treatment including sclerotherapy, excision, or another interventional trial; having plans for other treatments within the study period; allergy to the stocking material; regular use of diuretic medicine; clinical history of heart failure in the prior year; and other health or social conditions incompatible with this trial.

### 2.2. Sample Size

A prior pilot study of 13 KTS patients using custom-made compression garments for 4 weeks demonstrated a 3.0% mean difference (SD: 5.0) in limb circumference ratio. A one-sided paired *t*-test with alpha = 0.05 and 80% power determined that 15 subjects would be required. To accommodate potential dropouts, a sample size of 20 patients was targeted.

### 2.3. Protocol

After obtaining informed consent, baseline evaluations were performed and limb measurements were taken to fabricate custom-made elastic garments. Patients who had previously used compression devices were instructed to discontinue use at least three days prior to baseline assessment. No edema-reducing measures (e.g., limb elevation or compression) were used during this period.

The custom-made garments were produced by THUASNE (France) to deliver consistent 30 mmHg compression across the lower limb. Garment length varied based on lesion extent, with some extending to the buttocks. All garments reached at least the knee. Patients were instructed to wear the garment during waking hours, excluding bathing and sleep. Assessments were conducted before and after 26 weeks of treatment.

### 2.4. Outcomes

**Figure 1:**
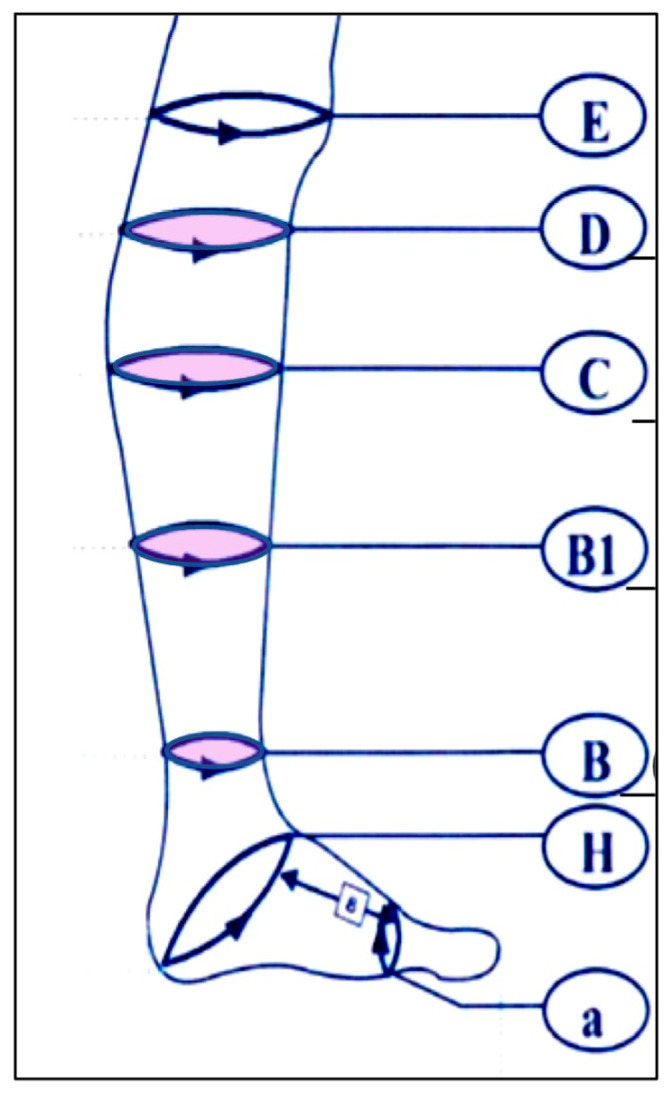
Measurement points for medical stockings. E: the knee joint perimeter, D: superior end of the tibial tuberosity, C: thickest part of the lower leg, B1: junction of the calcaneal tendon and gastrocnemius muscle, B: thinnest part of the ankle, H: from the instep to the heel, a: anterior part of the foot.

The primary endpoint was the change in circumference ratio (affected to unaffected limb) at four anatomical locations: the thinnest part of the ankle (B), junction of the calcaneal tendon and gastrocnemius muscle (B1), thickest part of the lower leg (C), and superior end of the tibial tuberosity (D). The ratios at each point were compared between baseline and week 26 (Figure 1).

Secondary outcomes included changes in pain, mRS score, body water content, vital signs (blood pressure, pulse), garment elasticity, and adverse events. Pain was assessed using patient/guardian questionnaires and clinician ratings at the beginning and the end of the study on a 1–5 scale (1 = no pain; 5 = severe pain). The questionnaire items included garment discomfort, pain severity, difficulty donning/removing the garment, activity restrictions (e.g., bathing, toileting), and symptoms such as wounds, bleeding, lymphorrhea, or infection.

Partial body water content was evaluated using bioelectrical impedance analysis (InBody S10, InBody Japan, Tokyo, Japan) at baseline and after 26 weeks. Measurements were taken 10–30 min after garment removal. Vital signs were measured on Day 1 (pre- and 10–30 min post-application) and the final day (pre- and post-removal).

Garment elasticity was measured by stretching two fixed garment points to a standard distance using a digital scale before use and at 26 weeks.

Adverse events were documented throughout the study period.

### 2.5. Statistical Analysis

The primary outcome data were analyzed using one-sided paired *t*-tests (significance: *p* < 0.05). Two-sided *t*-tests were used for water content, vital signs, and garment elasticity. Ordinal outcomes (pain and mRS) were analyzed with Wilcoxon signed-rank tests (*p* < 0.05). Analyses were conducted using IBM SPSS Statistics version 23.0 (IBM Corp., Armonk, NY, USA).

## 3. Results

This study included 7 male and 13 female patients aged 2 to 54 years (mean: 10.9 years). All participants successfully wore their custom-made elastic garment for the full 6-month study period. The mean height gain among the participants was 3.26 cm, and the mean weight gain was 1.65 kg. Table 2 summarizes the mean changes in limb circumference for both the affected and unaffected sides.

**Figure 2:**
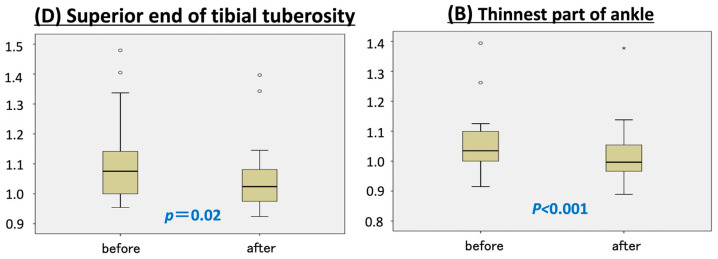
Circumference ratio change at measurement points B and D.

At the superior end of the tibial tuberosity (point D), the affected limb decreased by 4 mm in circumference, while the unaffected limb increased by 7 mm compared to baseline, resulting in a statistically significant decrease in the circumference ratio (*p* = 0.02) (Figure 2).

**Figure 3:**
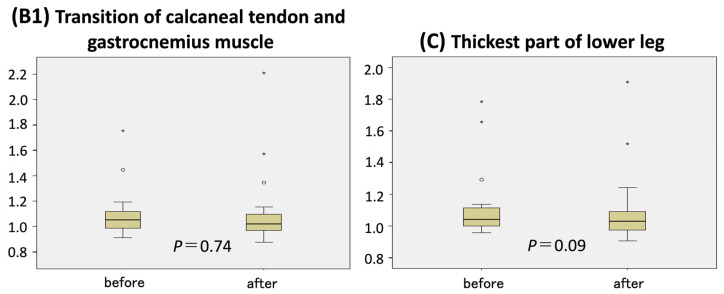
Circumference ratio changes at measurement points B1 and C.

Similar results were observed at the thinnest part of the ankle (point B), where the affected limb became 5 mm thinner and the healthy limb increased in size by 2.5 mm (*p* < 0.001). No significant changes in circumference ratio were observed at the remaining measurement points—B1 (junction of the calcaneal tendon and gastrocnemius) and C (thickest part of the lower leg) (Figure 3).

Pain and mRS scores showed a trend toward improvement after 6 months of compression therapy. Pain while wearing the garment decreased from a baseline mean of 1.7 to 1.5; pain without the garment declined from 1.5 to 1.44. The average mRS score improved from 1.6 to 1.4. Although some participants reported minor discomfort, such as a sensation of heat or itchiness, no restrictions in daily activities were noted.

No statistically significant differences in total or extracellular water content were observed between the baseline and post-treatment values in either the affected or healthy limbs, though all values showed a downward trend (Table 3).

Vital signs did not vary significantly throughout the program (Table 4).

**Figure 4:**
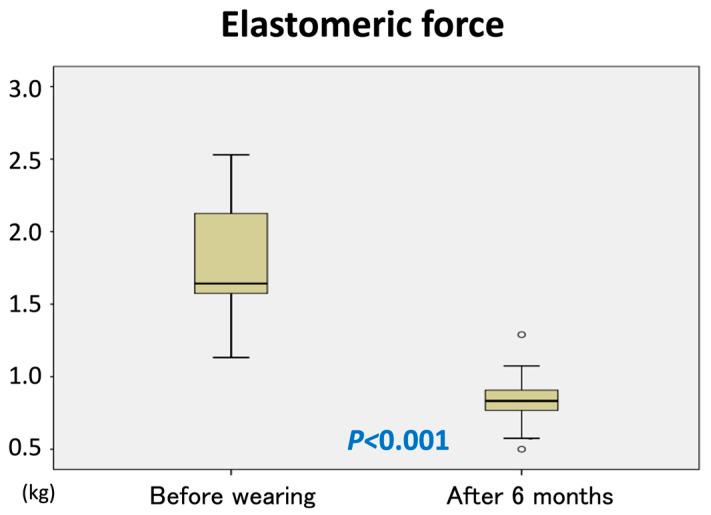
Change in elasticity force.

The elastic force of the garments significantly declined, measuring less than 50% of the initial force after 6 months of use (*p* < 0.001) (Figure 4).

No severe adverse events related to garment use were recorded. One patient developed a bacterial infection requiring hospitalization during the study period, but the infection site was outside the area of garment application, and a causal relationship was excluded. Another participant initially experienced difficulty donning the garment, which was resolved by fabricating a new garment. This participant successfully completed the study.

Overall, the use of the custom-made compression garments was well-tolerated and did not exacerbate any subjective symptoms.

## 4. Discussion

### 4.1. Initial Prospective Interventional Trial in Vascular Malformation Patients

Although several reports have described the effectiveness of compression therapy in alleviating vascular malformation symptoms, most have been retrospective and lacked robust evidence [7,8,9]. Previous studies on elastic garments primarily focused on venous disorders, such as post-thrombotic syndrome following deep vein thrombosis. One randomized controlled trial showed that wearing elastic stockings for two years significantly reduced symptoms such as edema, pain, and ulceration [10]. Another study involving 112 patients with chronic venous disease (CEAP classification C0–C6) demonstrated improvements in pain, swelling, pigmentation, physical activity, and overall health status after 16 months of using 35–40 mmHg compression stockings [11]. However, evidence remains limited regarding the efficacy of compression therapy in treating varicose veins without active or healed ulcers—conditions that share hemodynamic characteristics with VMs [12,13]. Our study is a prospective, interventional investigation that objectively demonstrates the efficacy and safety of custom-made elastic garments in patients with vascular abnormalities.

### 4.2. Effectiveness According to Limb Thickness

This study found that six months of wearing a custom-made 30 mmHg compression garment significantly reduced limb circumference at two measurement points: the superior end of the tibial tuberosity (point D) and the thinnest part of the ankle (point B). Of the four measured sites, the thickest part of the lower leg (point C) showed the least improvement. These findings suggest that compression therapy may be more effective in thinner regions of the limb.

According to Laplace’s law (*p* = T/r), where *p* is pressure, T is tension, and r is the radius, constant tension produces greater pressure in areas with smaller radii. Although the garments were designed to apply uniform tension, thicker regions such as point C likely received lower pressure compared to thinner regions. These results imply that custom garments with variable pressure distribution tailored to limb geometry may enhance their therapeutic effect in areas with greater girth. In this regard, the adaptation of Velcro-type garments and combinations of bandages and stockings may be effective, and testing the efficacy of these various compression therapies is a topic for future study.

### 4.3. Compression Therapy from Infancy and Childhood

KTS is often diagnosed at birth or in early childhood due to visible skin malformations and limb overgrowth [14,15]. In this study, the effectiveness of therapy was evaluated based on the circumference ratio between the affected and unaffected limbs, assuming symmetric natural growth.

**Figure 5:**
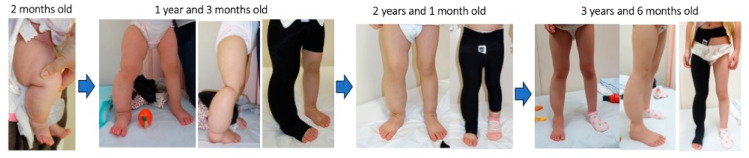
Representative clinical course of compression therapy from infancy.

A representative case (Figure 5) showed that the consistent use of a custom-made elastic garment from early childhood led to long-term symptom control with minimal invasiveness. To optimize outcomes and ensure adherence, it is important to minimize discomfort and daily activity restrictions. Although some participants reported sensations of heat or itching, none discontinued therapy, indicating that the garment was generally well-tolerated.

### 4.4. Pressure Retention and Durability

Compression pressure classifications vary by country. In Japan, compression is categorized as light (18–21 mmHg), weak (23–32 mmHg), medium (34–46 mmHg), and strong (>49 mmHg). The required pressure for venous compression varies by body position: 20–25 mmHg supine, 35–40 mmHg upright, and >60 mmHg for vein occlusion [16]. Although no universal compression standard exists for vascular anomalies, the prior literature recommends 20–30 mmHg for simple varicose veins (CEAP C2) and 30–40 mmHg for venous ulcers [6,17,18]. Our study used garments applying 30 mmHg of compression. However, the garment’s elastic force decreased by more than half over six months, suggesting that replacement at least twice annually is needed to maintain therapeutic benefit. The exact timing of elasticity loss remains uncertain and requires further study.

### 4.5. Thrombosis in Vascular Malformations

Vascular malformations are associated with localized intravascular coagulopathy, which can lead to thrombosis in some patients. VMs may enlarge over time and cause disfigurement or pain, often due to thrombotic events resulting in phlebolith formation [19]. Approximately one-third of pediatric VM patients exhibit elevated D-dimer levels due to localized coagulopathy, particularly in large or multifocal lesions, lesions with phleboliths, or cases of KTS with VM [20]. Mixed-type vascular malformations, such as in KTS, are also linked to a higher incidence of deep vein thrombosis and elevated D-dimer levels due to mild coagulopathy and low-flow dynamics [21].

Compression therapy may mitigate local coagulopathy in KTS by reducing blood stasis in addition to its mechanical slimming effects. However, the impact of compression therapy on thrombosis and coagulation parameters in VM remains unclear. Future investigations should incorporate serial coagulation testing to clarify these effects.

### 4.6. Limitations

This study was limited by its single-arm design and lack of a control group. Additionally, daily wearing time was not rigorously documented, which may have influenced the outcomes. The relatively small sample size also limits generalizability. It is also desirable to conduct separate studies on pediatric and adult patients. Future studies with larger cohorts and more comprehensive evaluations of coagulation markers are warranted.

## 5. Conclusions

Six months of wearing a custom-made 30 mmHg elastic garment significantly reduced limb circumference at two measurement points in patients with KTS and VM, without any severe adverse events. Given the observed decline in garment elasticity, replacement is recommended at least every six months to maintain therapeutic efficacy.

## Figures and Tables

**Table 1:** Essential diagnostic criteria for Klippel–Trenaunay syndrome.

**Essential findings for diagnosis**
Congenital mixed vascular malformations affecting nearly the entire area of one limb. The unilateral hypertrophy of soft tissue and/or bone on the same or opposite side of the affected limb.
**Exclusion criteria**
Sharply defined capillary malformation without evidence of deep vascular involvement. The simple thickening of extremities caused by isolated deep vascular malformations. Acquired vascular conditions, such as primary varicose veins or secondary lymphedema.
**Physical findings**
Lesions typically appear as masses or varicose-vein-like structures with bluish discoloration. Compression or elevation may cause partial collapse, while dependent positioning may result in swelling. These features may be less apparent in cases with significant thrombotic tendencies.
**Image findings**
Enlarged or clustered venous lumens with lobulated, spongy, or varicose morphologies identified via ultrasound, MRI, angiography, or contrast-enhanced CT. Slow blood flow may be present, with associated emboli or calcifications.

Translated from Japanese by M.N.

**Table 2:** Circumference changes at measurement points.

Average Change in Circumference	B		B1		C		D	
	Before	After	Before	After	Before	After	Before	After
**Affected limb**	17.47 cm	16.98 cm	23.91 cm	23.80 cm	28.13 cm	27.86 cm	26.43 cm	26.01 cm
	▼5 mm decrease		▼1 mm decrease		▼3 mm decrease		▼4 mm decrease	
**Healthy limb**	16.45 cm	16.69 cm	22.11 cm	21.89 cm	25.40 cm	25.73 cm	23.91 cm	24.66 cm
	▲2.5 mm increase		▼2 mm decrease		▲3 mm increase		▲7 mm increase	

**Table 3:** Changes in bioelectrical impedance analysis results.

Average Amount	Extracellular Water Content		Total Water Content	
	Before Trial	After Trial	Before Trial	After Trial
**Affected limb**	1.137	1.092	2.966	2.893
**Healthy side**	1.032	1.000	2.758	2.717

**Table 4:** Changes in vital signs.

Average	sBP	dBP	PR
** Before trial**			
** Before wearing**	104.8	63.5	93.6
** After wearing**	103.0	67.1	89.7
** After trial**			
** After wearing**	104.0	63.3	91.7
** After undressing**	102.2	64.2	93.3

Abbreviations: sBP, systolic blood pressure; dBP, diastolic blood pressure; PR, pulse rate.

## Data Availability

The original contributions presented in this study are included in the article. Further inquiries can be directed to the corresponding author.

## Abbreviations

The following abbreviations are used in this manuscript:

KTS	Klippel–Trenaunay syndrome
VM	Venous malformation
